# Mechanical Properties of Polypropylene-Based Flame Retardant Composites by Surface Modification of Flame Retardants

**DOI:** 10.3390/polym14173524

**Published:** 2022-08-27

**Authors:** Jinwoo Lee, Jae Hyung Park, Seung Bo Shim, Ji Eun Lee

**Affiliations:** 1 Korea Institute of Footwear and Leather Technology, 152 Dangamseo-ro, Busanjin-gu, Busan 47154, Korea; 2 Pusan National University, 2, Busandaehak-ro 63beon-gil, Geumjeong-gu, Busan 46241, Korea

**Keywords:** flame retardant, fire retardant, inorganic, phosphorus, resin blends

## Abstract

A flame retardant refers to a substance that can be added to a material having the property of being efficiently combusted to improve the material physically and chemically. It should not affect the physical properties required for the final product. Halogen-based compounds are representative flame retardants with excellent flame retardancy. However, their use is limited due to restrictions on the use of chemicals introduced due to human safety. Magnesium hydroxide, one alternative material of halogen flame retardants, is widely used as an eco-friendly flame retardant. However, the most significant disadvantage is high load. To find a solution to this problem, many studies have been conducted by mixing magnesium hydroxide with other additives to create a synergistic effect. In this study, flame retardancy and mechanical properties of polypropylene-based flame retardant composites as a function of mixing surface-modified magnesium hydroxide with phosphorus-based flame retardants were investigated. All materials including PP, additives, and flame retardants were mixed using an extrusion process. Specimens were prepared by an injection process of the compound made after mixing. As a result of the evaluation of the mechanical properties by the modified flame retardant, the relational expression of the mechanical performance degradation as a function of the amount of addition was obtained, and the tensile (CBATS) and bending strength (CBABS) were performed on the amount of flame retardant added. The relational expression obtained in this study is considered to be a formula for predicting the strength reduction according to the addition amount of the modified flame retardant and can be used in industry. In addition, it was found that the addition amount of the modified flame retardant had a greater effect on the lowering of the bending strength.

## 1. Introduction

Flame retardancy can be achieved by stopping the combustion process at one of its stages, where the process can be terminated before actual ignition occurs. The fastest method used to achieve flame retardancy is to incorporate flame retardants that may interfere with combustion at certain stages of the process so that the resulting system exhibits satisfactory flame retardancy. A polymer material is an organic material mainly composed of carbon, oxygen, and hydrogen atoms. It is a very combustible material. A flame retardant refers to a substance that can prevent the spread of combustion to a material such as a polymer with easy-to-combust properties by adding halogen, phosphorus, nitrogen, and a compound with a large flame retardant effect to improve the material physically and chemically. Mixability with raw materials and additives should be excellent. The flame retardant should not affect properties required for the final product [1,2]. Constituents of the flame retardant can be divided into organic and inorganic types. Organic retardants are represented by nitrogen, halogen, and phosphorus. Inorganic flame retardants are divided into antimony types and metal hydroxide. Halogen-based compounds, which are representative flame retardants, have the advantage of providing excellent flame retardancy regardless of the applied polymer. However, the disadvantage is that the amount of gas generated during combustion of the compound is relatively large compared to other flame retardants. This is because the compound is decomposed to generate halogen radicals when burning. Radicals can capture hydrogen or hydroxy radicals that affect combustion, thus interfering with the combustion process and leading to a flame retardant effect in the gas phase. In addition, a lot of corrosive hydrogen halide is emitted during the radical trapping process, and this gas is a toxic gas that is harmful to the human body. Due to increased environmental awareness of the entire chemical industry, strict laws on additives and regulations are gradually improving. New regulations should lead to halogen-free flame retardants with lower toxicity, flammability, improved stability, and flame retardant properties [3,4,5].

Halogen-free flame retardants are mainly inorganic hydroxides, foaming flame retardants, nano-sized flame retardants, and fluorescence-containing flame retardants. Among them, magnesium hydroxide generates H_2_O during combustion and turns into water vapor, diluting the combustible gas to lower the temperature around the combustion point, suppressing the combustion phenomenon, and acting as an effective smoke suppressant. It is also widely used in industrial applications due to its abundant source, relatively high decomposition temperature, low toxicity, and thermal insulation effects [6,7,8,9,10,11,12,13].

Magnesium hydroxide, as one of inorganic hydroxides, is an eco-friendly flame retardant that can simultaneously improve flame retardancy and smoke suppression. It is widely used in industrial applications due to its low cost, high thermal stability, low toxicity, and thermal insulation effects. In particular, it is one of the most eco-friendly flame retardants because only water vapor is emitted during combustion. In addition to its ability to inhibit polymer ignition, it can also act as an effective smoke suppressant [14,15,16]. However, since the high load of magnesium hydroxide is known as a disadvantage, many studies have been performed by mixing magnesium hydroxide with other additives to create a synergistic effect. It has been shown that the addition of high levels of magnesium hydroxide filler can readily affect the mechanical properties of composite materials [7]. Jiao et al. [17] studied the effects of magnesium hydroxide on flame retardancy and mechanical properties of EVA/MH composites through the limiting oxygen index (LOI), the cone calorimeter test (CCT), and tensile tests. It was confirmed that magnesium hydroxide prepared in an alkaline environment was effective in improving the flame retardancy of ethylene-vinyl acetate (EVA).

Polypropylene (PP) has many uses worldwide. PP is useful in many applications such as textiles, films, filaments and injection molded parts in automobiles, rigid packaging, consumer electronics, medical devices, food packaging, and consumer goods. PP can easily deteriorate during processing due to excessive thermal oxidation [18]. Because PP is easily flammable with a low limiting oxygen index of around 18%, its use in electrical and electronic materials is limited. Reinforcing agents and flame retardants are added to PP to improve the flame retardancy of polypropylene [19,20].

The type of flame retardant and the amount needed to achieve a specific purpose will depend on the specific polymer. Various halogen or phosphorus-containing flame retardants [21], such as metal hydroxides and oxides [5], nanocomposites [22], and intumescent [23] systems, are usually applied synergistically to PP composites, polypropylene and epoxy resins [13,24,25,26,27,28,29] to improve flame-retardant efficiency [15]. It was found that the flame retardancy of the polymer was affected by the type and content of the flame retardant [30].

Today, phosphorus-based flame retardants (FRs) are considered the best alternatives to toxic halogen additives. Phosphorus FRs can act in the condensed phase (by enabling the creation of crosslinked and carbonized structures, especially in polymers containing oxygen) or in the gas phase (by removing the high-energy radicals H and OH thanks to the formation of active radicals. The use of phosphorus-based FR additives and inorganic additives [31] such as ammonium polyphosphate (APP) [16], talc [32], and montmorillonite (MMT) [31], alone or simultaneously [33], as a solution to the low mechanical and flame retardancy of PP is recognized. APP is a stable non-volatile compound. Upon contact with water, APP slowly hydrolyzes to monoammonium phosphate (orthophosphoric acid). The hydrolysis process can be accelerated by prolonged exposure to higher temperatures and water. Long-chain APP begins to decompose into poly(phosphoric acid) and ammonia at temperatures above 300 ℃. Short-chain APP begins to decompose at temperatures above 150 °C. APP and APP-based systems are highly efficient halogen-free flame retardants. APP is a non-toxic, eco-friendly material and does not produce any additional smoke due to its unique expansion mechanism. Compared to other halogen-free systems, APP requires a lower load [30].

Tang et al. elaborated the mechanical combustion of a PP/PA compatible blend containing APP and MMT, highlighted the flame retardant effect of PA on PP, and reported a synergistic FR function between APP and MMT [34,35]. Kinematic studies performed by Almeras et al. confirmed the FR role of PA on the flame retardancy of PP in PA/PP blends containing poly(vinyl alcohol-co-ethylene) copolymer as an APP FR additive and coupling agent [35,36]. Ma et al. reported similar results for carboxylated PP and compatibilized PP/PA blends in the presence of organically modified MMT via morphology, calorimetry, rheology, and charcoal content analysis [37].

However, studies have found acceptable resistance to fire and pyrolysis in systems containing one type of FR. Magnesium hydroxide must use high loads of up to 50%, which is sometimes detrimental to the mechanical properties [38]. Adding large amounts of flame retardants can severely degrade the properties of thermoplastics and may cause processing problems. Flame retardants (FRs) that plasticize polymers reduce thermal properties such as heat deflection temperature, while insoluble solid additives can severely degrade impact properties.

In this study, experiments were conducted using inorganic flame retardants such as magnesium hydroxide and phosphorus flame retardants as main raw materials. In order to overcome the decrease in mechanical strength due to the increase in the amount of the flame retardant used, surface treatment was performed on magnesium hydroxide, and changes of the mechanical properties by the amount used were observed. According to the results of a number of existing researchers, the degree of improvement in flame retardant properties was insufficient, with LOI ranging from 18 to 23, even though the content of APP flame retardant alone was used up to 30% [39,40,41,42,43,44,45,46,47,48,49,50,51,52,53,54]. In addition, a compound was prepared by mixing modified magnesium hydroxide and phosphorus-based flame retardant to reduce the amount of flame retardant used overall and to improve flame retardant properties. Flame retardant properties and mechanical properties of each composition were evaluated.

## 2. Materials and Methods

### 2.1. Specimen Preparation

In consideration of economic feasibility and flame retardant performance, experiments were conducted using inorganic flame retardants (magnesium hydroxide; M-soluchem, Cheonan-si, Korea) and phosphorus flame retardants (APP, ammonium polyphosphate; M-soluchem, Cheonan-si, Korea) as main raw materials. Inorganic flame retardants were used by pulverizing inorganic particles to increase compatibility with resins, minimize deterioration of polymer resin properties, and maximize flame retardant effects. A product having a particle size of 42 μm (mean) of magnesium hydroxide was used in the experiment. In general, the more the material is micronized, the better the flame retardant performance. To control the effect of micronization on flame retardant performance, a flame retardant having a uniform particle size of 11 μm (mean) was obtained through a primary micronization process. In addition, in order to increase the compatibility of an inorganic flame retardant with resin and improve flame retardant effects and intrinsic viscosity, inorganic particles were surface-modified with a silane (M-soluchem, Cheonan-si, Korea) and melamine-based compounds to prepare an inorganic composite flame retardant.

The dispersibility and flame retardant effect were improved by surface-modifying silane on the surface of the inorganic flame retardant. As a surface modifier, N-octyltriethoxysilane (MSi; M-soluchem, Cheonan-si, Korea) was used. EtOH (M-soluchem, Cheonan-si, Korea), inorganic particles, and silane were put in a flask with a reflux cooling device. The wet method was reformed by stirring at 400 rpm while purging N_2_. The dry method of reforming used a ball milling machine (Hankook E.M, Pheongtaek-si, Korea) to modify the surface.

In the wet reforming process, the ratio of ethanol (M-soluchem, Cheonan, Korea) to deionized water was 80:20 at atmospheric pressure to make a mother solution. The pH was adjusted to 4.5 using acetic acid (M-soluchem, Cheonan, Korea) with stirring. While stirring, 1–3 phr of the silane modifier was added. After stirring at 400 rpm, a one-step hydrolysis reaction was performed. After the hydrolysis reaction was completed, an inorganic flame retardant was added, and the surface modification reaction was performed at 300 rpm, followed by filtering. It was prepared by washing three times using ethanol/deionized water (80:20) solution, washing with pure ethanol, and drying in a dry oven(Hankook E.M, Pheongtaek-si, Korea) at 150 °C for 6 h.

The base polymer used for the test was Korea Perrochemical Ind. Co., Ltd.’s Poly-pro4018, melt index of 19 g/10 min, density of 0.9 g/cm^3^, and melting point of 167 °C, general homo PP. The mixing ratio of the polymer and the flame retardant is shown in Table 1, and the flame retardant was added in a certain proportion by weight based on 100 g of the base polymer. Specimens for the evaluation of resin properties by Mg(OH)_2_ with surface modification were manufactured in the form of a 3 mm thick flat plate with a 5 ton hot-press (HPS-150, Ilshin autoclave Co., Ltd., Daejeon, Korea) after making a compound using a twin screw extruder (Hankook E.M, Pheongtaek-si, Korea). A flat plate was manufactured by applying a compression load of 15 MPa for 10 min at a molding temperature of 210 degrees. Cooling time was 10 min or more at room temperature. The screw was divided into 7 sections, and the temperature was controlled for each in a twin screw extruder. The temperatures in the seven sections from the extrusion die to the screw feeding the material were 200, 210, 190, 180, 170, 160, and 150 °C, respectively. The L/D ratio of the equipment thread was 40, the rotation speed was 40 rpm, the feed screw speed was 12–15 rpm, and the feed rate was about 5 kg/h.

A test piece for evaluation of the mechanical properties of a composite material with various addition and blending of flame retardants was manufactured using an injection molding machine (Dong-Shin Korea, Changwon-si, Korea).

Injection was carried out at 220 °C for 3 s, and the temperature of the mold was 50 °C. Injection pressure and holding pressure were set to 1625 bar, and injection and holding pressure were maintained for 3 s and 5 s, respectively. After injection, the mold was demolded with a cooling time of 30 s.

### 2.2. Test Methods

The tensile strength of the propylene-based flame retardant composite material was measured at room temperature using a universal test machine (DUT-500CM, Daekyung Engineering, Busan, Korea) according to ASTM D638. A 50 kN cell was used for the load cell (Daekyung Engineering, Busan, Korea). The crosshead speed was measured at a speed of 50 mm/min.

Bending strength was measured using the same equipment as tensile strength according to the ASTM D790 standard. The measurement speed was measured at a speed of 30 mm/min. Impact strength was measured using an Izod impact tester (Daekyung Engineering, Busan, Korea) with a notched specimen at room temperature in accordance with the standards of ASTM D256.

To evaluate the injection moldability of the modified Mg(OH)_2_, the melt flow index was measured according to ASTM D1238. The equipment used was M.D.R. (DST, Busan, Korea), and the load applied in the experiment was 2.16 kg at a temperature of 230 °C.

Limiting oxygen index (LOI) refers to the volume percentage of the least oxygen in oxygen-nitrogen mixed air required for a polymer sample to be ignited and burn without being extinguished for 3 min. The limiting oxygen index measurement method was developed by General Electronic in 1957 to measure the combustibility of fuel. Because of the excellent reproducibility of the experiment and easy product quality control, it is a combustibility test method for polymer products that is used in many countries. It has been adopted as a standard [19]. The test apparatus consists of an igniter part, a gas supply part composed of oxygen and nitrogen gas cylinders, a combustion part including a heat-resistant glass combustion cylinder (DST, Busan, Korea), and a measuring part including a flow meter and a gas mixer (DST, Busan, Korea). At a constant flow rate, the sample hung vertically from the clamp of the sample located at a position of 100 mm or more above the combustion cylinder is ignited and burned. The minimum flow rate of oxygen and nitrogen required for the combustion time of the sample to continue for more than 3 min or for the innate combustion field to continue combustion for more than 50 mm is determined when the sample is on fire. If the oxygen flow rate at that time is [O_2_] (L/min) and the nitrogen flow rate corresponding to this flow rate is [N_2_] (L/min), the oxygen index is OI(O.I.) = [O_2_] × 100/([O_2_] + [N_2_]). The larger the OI (O.I.) value, the greater the flame retardancy. In this study, the flame retardancy of each polypropylene-based flame retardant composite material was comparatively evaluated using LOI.

## 3. Results

**Figure 1:**
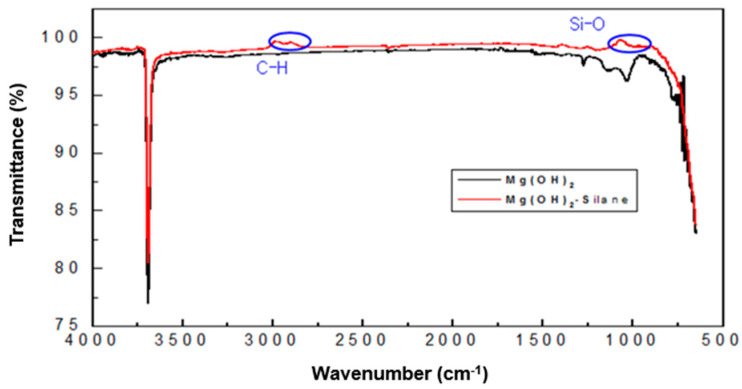
FT-IR spectra of Mg(OH)_2_ and Mg(OH)_2_ with silane.

**Figure 2:**
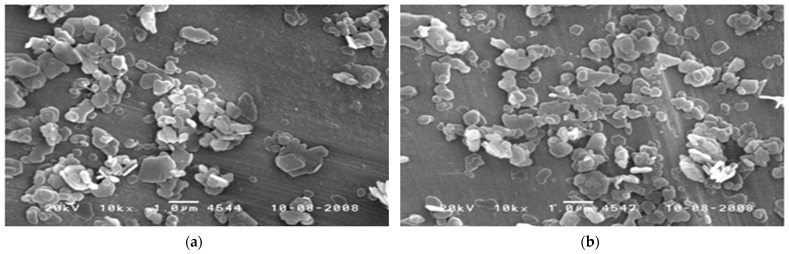
SEM observation results of magnesium hydroxide by surface treatment: (**a**) unmodified Mg(OH)_2_; (**b**) surface treatment with silane (MSi).

To minimize the flame retardant effect of the resin and the inorganic flame retardant and to determine the deterioration of physical properties of the polymer resin, inorganic particles were surface-modified with a silane (MSi) compound for the purpose of increasing compatibility. Figure 1 shows results of FT-IR and SEM analysis of magnesium hydroxide surface-modified with silane. The black line is the FT-IR result of magnesium hydroxide. The red line is the measurement result after surface treatment with a silane compound. It was confirmed that C–H stretching and Si–O stretching peaks, which are silane compound components that do not appear in the black line, appeared in the red line in the vicinity of 2900–3000 cm^−1^ and 1000 cm^−1^ of the FT-IR graph. It can be seen that in the sample before the reforming reaction, a characteristic peak due to the hydroxyl group (–OH) present in Mg(OH)_2_ was strongly observed near 3500–3750 cm^−1^ and between 3700 cm^−1^ and 3600 cm^−1^ during silane reforming. It can be seen that the characteristic peak of –OH is reduced because it is substituted by the reaction of the hydroxyl group present in Mg(OH)_2_ and the alkoxide group present in the silane. Figure 2 shows a photograph of magnesium hydroxide particles observed through SEM before and after reforming. It was confirmed that the surface-treated SEM image using MSi showed a relatively large particle size compared to untreated magnesium hydroxide. When looking at the surface of the sample before surface modification, an agglomerated Mg(OH)_2_ phenomenon was observed before surface treatment. After treatment with silane, it was judged to exhibit a more uniformly dissolved shape without a significant change in particle size, and it is thought that dispersibility will be improved upon melt mixing with PP.

**Figure 3:**
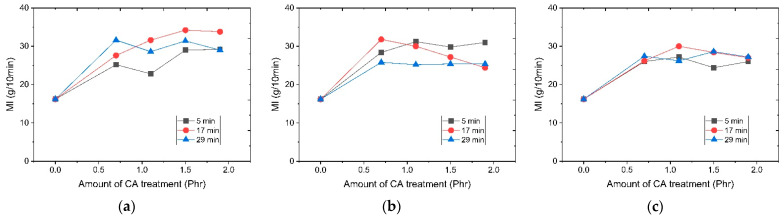
MI graph as a function of surface modification temperature and CA addition amount: (**a**) 130 °C; (**b**) 150 °C; (**c**) 170 °C.

**Figure 4:**
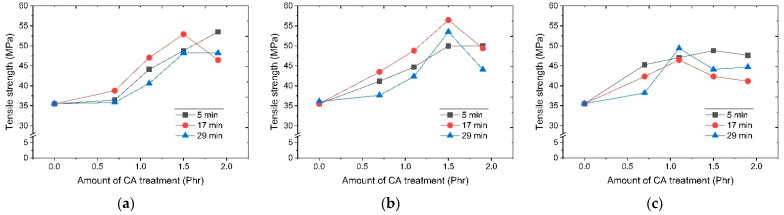
Tensile strength depending on surface modification temperature and CA addition amount: (**a**) 130 °C; (**b**) 150 °C; (**c**) 170 °C.

**Figure 5:**
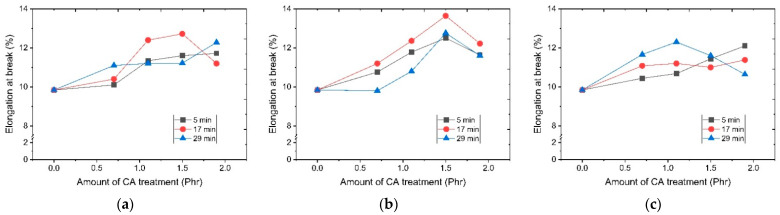
Strain rates depending on surface modification temperature and CA addition amount: (**a**) 130 °C; (**b**) 150 °C; (**c**) 170 °C.

In order to confirm changes in the physical properties of resin depending on dry surface processing temperature, the melt flow index and the change in tensile strength and elongation at break as function of a surface modification temperature and time were divided into modifier usage and resin dispersion processing conditions. Results are shown in Figure 3, Figure 4 and Figure 5. It was confirmed that the tensile strength and elongation were increased when a CA (coupling agent) was used in the overall test piece. At a surface modification temperature of 130 °C, the melt flow index generally increased as a function of surface treatment of the silane modifier. Tensile strength and elongation showed a tendency to increase with an increasing amount of modifier used at the treatment times of 5 min and 29 min. However, at 17 min after 1.5 phr of modifier usage, both elongation and tensile strength decreased. At a surface modification temperature of 150 °C, the change of the melt flow index showed a similar tendency to the result at 130 °C. However, tensile strength and elongation showed the maximum value at 1.5 phr of the CA modifier. Thereafter, it showed a tendency to decrease. It is presumed that if the surface treatment time is too short, the surface treatment will be incomplete. If it is too long, functional deterioration due to excessive reaction will occur. If the amount of the surface treatment agent is too small, an untreated surface will result. If the amount of the surface treatment agent is too large, it is thought that the excess CA will deteriorate the resin properties. At 170 °C, it was confirmed that the trend was different from the trend at the previous two temperatures. The tensile strength and elongation were low overall.

**Figure 6:**
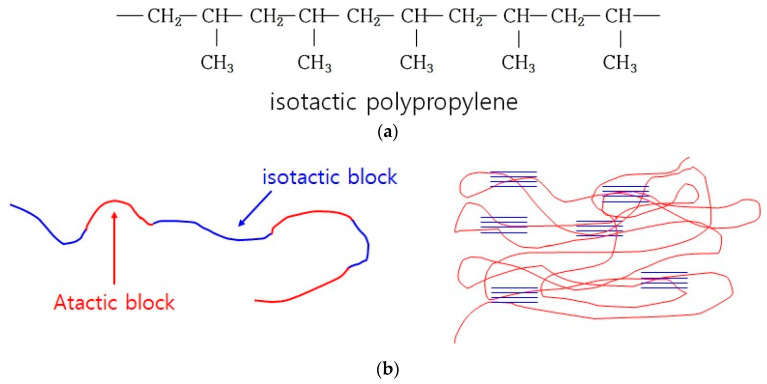
Schematic diagram of the combination of isotactic and atactic structures: (**a**) isotactic polypropylene structural formula; (**b**) structural formula consisting of atactic and isotactic single bonds (left), and structural formula of block copolymer in which many isotactic structures are formed and combined (right), Blue line is isostatic block, Red line is Atactic block.

In the case of surface treatment with CA, it was judged that the flame retardant particle size was uniform as confirmed in Figure 2. When it was dispersed in PP, it was judged that the degree of dispersion increased, and the overall flowability improved (Figure 3a). Therefore, transformation from the isotactic structure to the atactic structure occurred. Figure 6a shows the structure of isostatic polypropylene. Figure 6b is a schematic diagram of an elastomer in which crystalline and amorphous parts coexist (connection structure of atactic block and isotactic block). When a large amount of atactic structure is generated, it becomes an elastomer in which a crystalline part (blue, isotactic) and an amorphous part (red, atactic) coexist in the form of a block copolymer, and an increase in elongation is expected, but Since there is not much content, it is thought that only a slight atactic structure is generated, indicating a tendency for the elongation to decrease. Therefore, it is judged that the improvement effect of the tensile strength of the composition using the flame retardant surface treated at 150 degrees for 17 min is the greatest (Figure 4b).

Considering that the overall tensile strength and elongation were high when the treatment time was 17 min at 130 °C and 150 °C, it was presumed that if the surface treatment time was too short, the surface treatment would be incomplete. If it was too long, functional deterioration due to an excessive reaction would occur. It can be presumed that functional deterioration occurs even when the temperature is excessively high.

**Figure 7:**
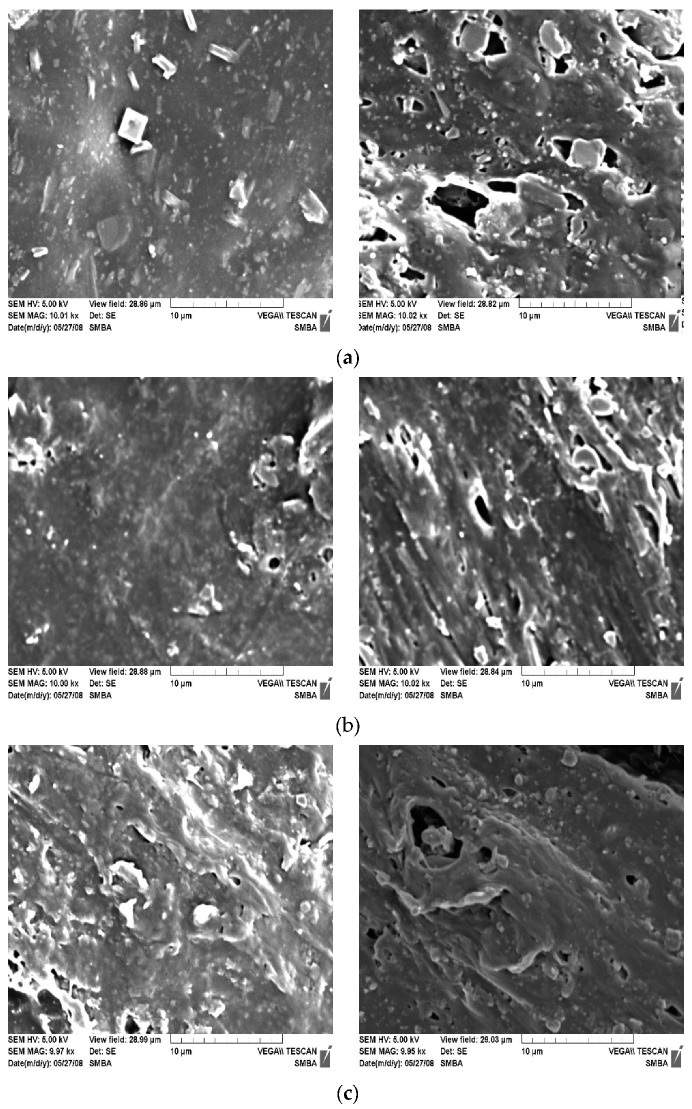
Comparison of SEM photos before and after tensile test of specimen surface (top), injection specimen (right), and surface immediately after tensile test: (**a**) Mg(OH)_2_ without treatment; (**b**) Mg(OH)_2_ with CA treatment; (**c**) Mg(OH)_2_ with excessive amount of CA.

Modified inorganic flame retardant surface properties were determined. Figure 7 shows SEM images of surfaces of specimens not treated with the CA modifier, a specimen treated with the CA modifier, and a specimen after the tensile test. The surface of the untreated Mg(OH)_2_ test piece was relatively rough. In the test piece after the tensile test, many fractures occurred due to tensile stress. Many holes were observed on the surface. It could be seen that the combination of the two did not occur smoothly. Contrary to this, the surface of the injection specimen treated with silane was relatively smooth. It was confirmed that the compound was in a good dispersion state. Mg(OH)_2_ and resin had a strong bond. On the other hand, in the specimen to which an excessive amount of CA was added, aggregated Mg(OH)_2_ was not dispersed in the resin. It existed as an impurity in the resin. Thus, the surface of the injection specimen was not uniform. As a result, it was confirmed that the physical properties were also lowered.

**Figure 8:**
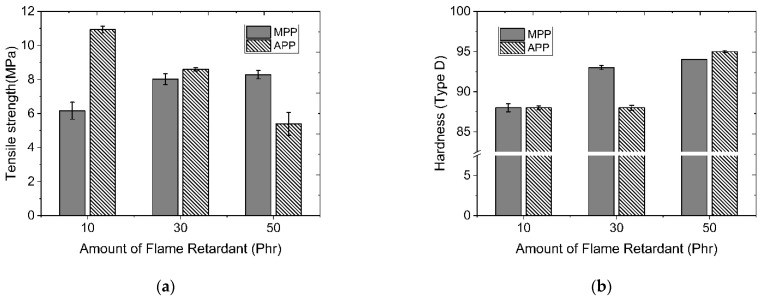
Comparison of physical properties depend on the flame retardant type (MPP, APP) and added contents: (**a**) tensile strength; (**b**) hardness.

In order to minimize the dry flame retardant content and increase the flame retardant effect, properties of each composition in which a melamine-based and phosphorus-based flame retardant was added as an additive to a single PP material in a range of 10 to 50 phr were evaluated separately from the previously modified material. Figure 8 shows a graph comparing tensile strength and hardness characteristics as a function of the type and content of flame retardants. In the case of MPP (melamine polyphosphate), the tensile strength showed a tendency to increase as a function of the addition of the flame retardant. However, the tensile strength of APP showed a tendency to decrease depending on the increase in the content of the flame retardant. The hardness as a function of the amount of the flame retardant added generally showed a tendency to increase as the content of the flame retardant increased. In MPP with a low initial strength, the tensile strength and hardness slightly increased with increasing addition of the flame retardant. The tensile strength also decreased due to an increase of the brittle fracture phenomenon.

**Figure 9:**
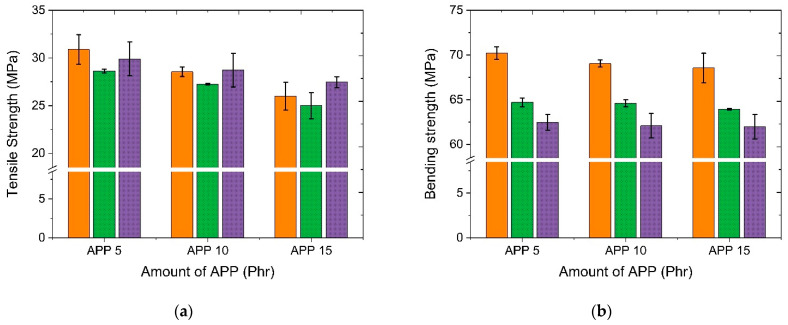
Comparison of physical properties as a function of the addition amount of APP flame retardant and modified flame retardants: (**a**) tensile strength; (**b**) hardness.

Compared to MPP, which had a phosphorus content of 12% to 14%, APP, which had a higher phosphorus content of 29% to 32%, showed relatively high tensile properties. It was judged to be advantageous in terms of flame retardancy evaluated. The APP content showed the highest tensile properties at 10 phr. Therefore, the mechanical properties were evaluated by changing the amount of APP added to 5, 10, and 15 phr and controlling the content of the modified flame retardant. Figure 9 shows tensile strength and bending strength characteristics as functions of modified flame retardant contents and APP flame retardant contents. As the content of flame retardant increased, the specific gravity of both APP and modified flame retardant increased. However, the hardness had little change. The tensile strength showed a tendency to decrease by the addition of APP flame retardant. The modified flame retardant showed the greatest decrease at 2.5 based on phr. It showed a higher tensile strength than 2.5 at 5 phr. As for the bending strength, it showed a tendency to decrease for both APP flame retardant and modified flame retardant as the amount of addition increased.

In order to evaluate the effect of the increase in the amount of APP flame retardant on the change in tensile strength and bending strength, the correlation between the increase in the amount of APP flame retardant and the amount of change in each mechanical property was evaluated. Equations (1) and (2) are equations that were used for calculating the correlation between the change in tensile strength and bending strength by the change in the amount of APP added, respectively. The relationship between the increase in the amount of APP and the tensile strength and bending strength was linearly obtained through trend line analysis of the graph. The slope value of a one-dimensional graph was obtained. The correlation between APP addition content change and tensile strength (CBATS), and the correlation between APP addition content change and bending strength (CBABS) were calculated with the following equations:

(1) $$\mathrm{CBATS}=\frac{\Delta \mathrm{APP},\mathrm{phr}}{\Delta \sigma \left(T.S\right)}$$

(2) $$\mathrm{CBABS}=\frac{\Delta \mathrm{APP},\mathrm{phr}}{\Delta \sigma \left(B.S\right)}$$

In the above formula, ΔAPP, phr is the change in the amount of flame retardant added, Δσ(T.S) is the change in tensile strength, and Δσ(B.S) is the change in bending strength.

**Figure 10:**
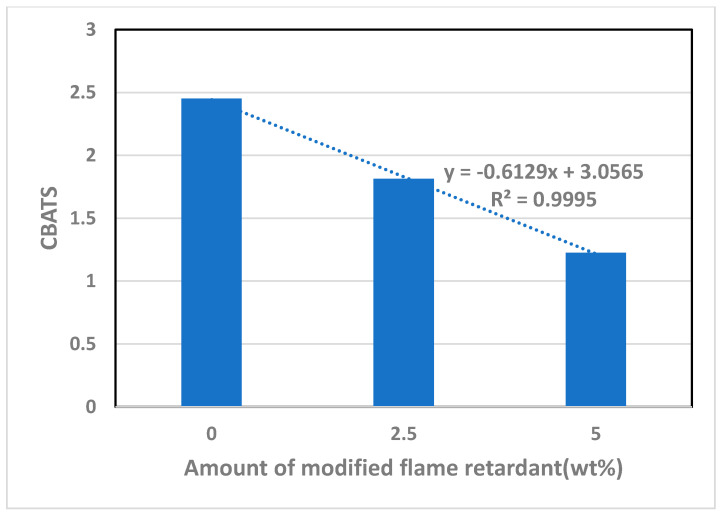
Relationship with CBATS as function of the increase in the amount of modified flame retardant added.

Figure 10 is a graph showing CBATS by the increase in the amount of the modified flame retardant. It could be seen that the CBATS showed an inversely proportional curve with the addition of modified flame retardant. Thus, it is possible to estimate the tendency of the tensile strength decrease based on the addition of the APP flame retardant as the added amount of the modified flame retardant increases. The amount of APP flame retardant and modified flame retardant can be considered as a factor directly affecting the decrease in tensile strength.

**Figure 11:**
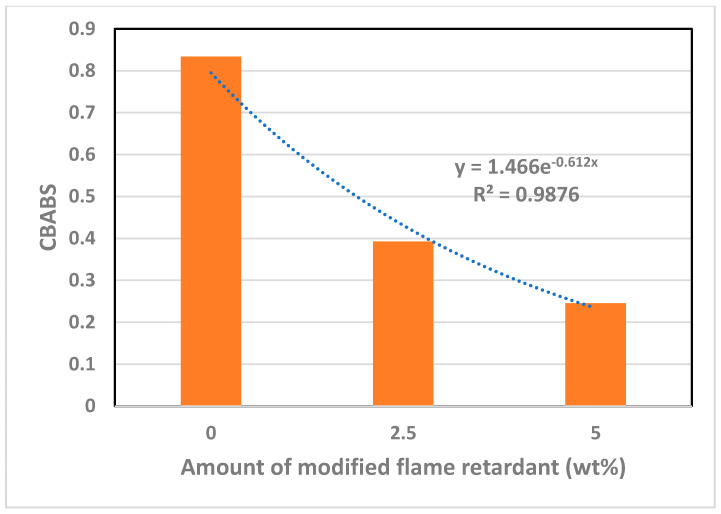
Relationship with CBABS by increasing the amount of modified flame retardant added.

On the other hand, it could be seen that CBABS (Figure 11) decreased exponentially with the addition of flame retardant. Through the two graphs, it can be considered that the increase in the amount of modified flame retardant had a greater effect on the reduction of bending strength than on tensile strength. It is considered that modified flame retardant should be added while paying attention to the bending strength required when designing flame retardant blends. The correlation between CBATS and CBABS and the modified flame retardant needs to be established through many additional studies. It can be used as a standard for flame retardant blending when a lot of data is accumulated.

In order to evaluate flame retardancy performance by injecting a compound containing an APP flame retardant and a modified flame retardant, an evaluation was conducted by performing a combustibility test and a limiting oxygen index (LOI) test according to the MS-300-08 test standard. The combustibility test was performed to burn within 60 s without burning more than 50 mm from the measurement point. All test pieces showed satisfactory performance.

**Figure 12:**
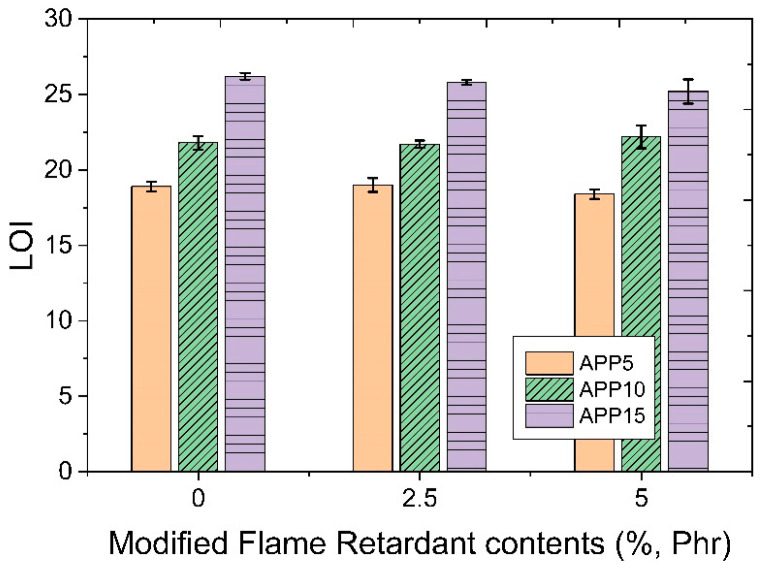
LOI measurement results as a function of the amount of modified flame retardant and APP added.

Figure 12 shows LOI evaluation results as a function of the addition amount of APP flame retardant and modified flame retardant. In marginal oxygen index evaluation, the lowest concentration of oxygen required for continuous combustion of a sample was ignited in an air stream mixed with oxygen and nitrogen. The higher the oxygen index, the better the flame retardancy of the material. When a polymer material has a value of 30 or higher, it is suggested that the material has flame retardancy [7]. In this study, the minimum oxygen index required for ignition and combustion was evaluated as a material that did not continue to burn or did not ignite when it was below 21%. When the content of APP was 10 or more in all specimens, a value satisfying the LOI evaluation was shown. Both the modified flame retardant and the APP flame retardant showed a tendency to increase in LOI with an increase in the amount added. Thus, it is thought that both flame retardants play a role in helping flame retardant performance. However, overall, the tensile strength and bending strength tended to decrease depending on the use of flame retardants.

**Figure 13:**
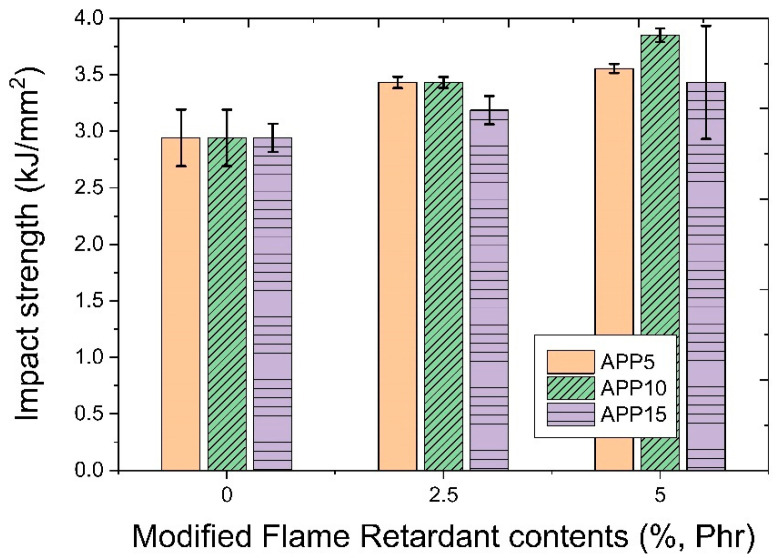
Impact strength characteristics as a function of the amount of modified flame retardant and APP added.

Figure 13 shows a graph presenting the impact strength characteristics as a function of the amount of modified flame retardant and APP added. After adding 5 phr, 10 phr, and 15 phr of APP flame retardant based on the base polymer, respectively, to the test piece to which the modified flame retardant was not added, there was no change in impact strength as a result of measurement of the prepared test piece. In the test piece with the modified flame retardant addition in the amount of 2.5 phr, the impact strength was increased in all test pieces to which the APP flame retardant was added compared to the unadded test piece. The amount of increase was relatively small for the test piece to which 15% of APP was added. The impact strength was increased even when the modified amount of addition of 5 phr in the test specimen was compared to the unadded test specimen. However, there was a difference in the amount of increase in impact strength depending on the amount of APP flame retardant added. Overall, the impact strength showed a tendency to increase with an increase in the amount of the modified flame retardant. The single addition of the APP flame retardant did not significantly affect the impact strength. It is considered that optimization of the amount of both flame retardants is necessary to obtain high impact strength by adding two flame retardants as test specimens containing both modified flame retardants and APP having the highest impact strength content.

Based on the same APP content, it is judged that the modification reaction caused by the silane coupling agent, which is a surface treatment agent of magnesium hydroxide, inhibits the β-scission reaction, as the impact strength increases as the content of the modified flame retardants increases. This is thought to improve the impact strength due to branching and crosslinking reactions. In addition, when evaluated based on the same modified flame retardant contents, the impact strength tends to decrease as the content of APP increases. It is considered that as the content increases, the polymer content decreases, and thus the overall molecular weight decreases, indicating a tendency to decrease the impact strength.

**Figure 14:**
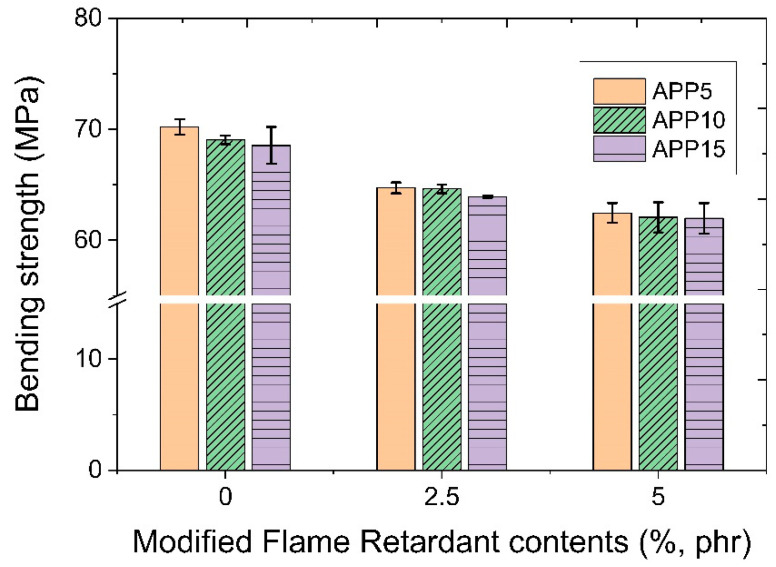
Bending strength characteristics as a function of the amount of modified flame retardant and APP added.

Figure 14 shows bending strength characteristics as a function of the addition amount of the modified flame retardant and the APP flame retardant. As for bending strength characteristics, it was found that bending strength decreased as the amount of the modified flame retardant and APP flame retardant increased. It is considered that the modified flame retardants present at the interface of the polypropylene polymer could act as a stress concentration part, which could decrease bending strength by decreasing the strength of the interface. When bending stress is applied, compressive stress is applied to the side where the stress is applied, and tensile stress is applied to the opposite side.

Since both APP and flame retardants have lower strength and hardness compared to PP, their use reduces the stiffness and toughness of the composition, resulting in a decrease in tensile strength and flexural strength.

Overall, the flame retardancy increased (LOI 16% or less – 26.2% increased) as the amount of flame retardant increased. The impact strength also showed a tendency to increase. The hardness did not change much. However, the specific gravity showed a tendency to increase with increasing flame retardant. With the use of flame retardants, tensile strength and flexural strength were lowered, making improvement necessary.

## 4. Conclusions

In this study, a particle-reinforced composite material was prepared by blending inorganic flame retardants, magnesium hydroxide and phosphorus flame retardants, into polypropylene. The following conclusions were obtained by evaluating the flame retardancy and mechanical properties of the composite material by the surface modification of the inorganic flame retardant and the type of flame retardant.

1. When specimens using surface-treated inorganic particles were arranged in a good dispersion state and in the direction in which stress was applied after tensioning, no holes were formed, confirming the strong bond formation state with the resin.
2. As a result of comparing tensile strength and hardness characteristics by the type and content of the flame retardant, in the case of MPP, the flame retardancy tended to increase as the amount of the flame retardant increased. However, tensile strength and flexural strength were lowered. Thus, additional research is needed for optimization and improvement.
3. CBATS decreased linearly, and CBABS decreased exponentially as a function of the amount of the modified flame retardant added. It is considered that the increase in the amount of modified flame retardant has a greater effect on bending strength than on tensile strength.
4. Through the investigation of the relationship between the modified flame retardant and the mechanical strength, it is thought that the utilization will be high as basic data for the compounding of the composite material that can satisfy the flame retardancy and mechanical strength required in the automobile industry. In particular, it is expected to expand to the application of backward injection materials for interior components such as door trims in the automobile industry, where the demands for eco-friendly production such as weight reduction and integration of production process are increasing.

## Figures and Tables

**Table 1:** Mixing ratio of various flame retardants added to compounds for composite material property evaluation.

Specimen Number		P1	P2	P3	P4	P5	P6	P7	P8	P9	P10	P11	P12	P13	P14	P15
Base Polymer	PP	100	100	100	100	100	100	100	100	100	100	-	-	-	-	-
Flame retardant (phr, part of a hundred resin)	MPP	10	30	50	-	-	-	-	-	-	-	-	-	-	-	-
	APP	-	-	-	10	30	50	5	10	15	5	10	15	5	10	15
	Modified Mg(OH)_2_	-	-	-	-	-	-	-	-	-	2.5	2.5	2.5	5	5	5

## Data Availability

Not applicable.
